# Parental migration and the mental health of those who stay behind to care for children in South-East Asia

**DOI:** 10.1016/j.socscimed.2014.10.060

**Published:** 2015-05

**Authors:** Elspeth Graham, Lucy P. Jordan, Brenda S.A. Yeoh

**Affiliations:** a Department of Geography and Sustainable Development, University of St Andrews, St Andrews, Fife KY16 9AL, UK; b Department of Social Work and Social Administration, 5/F The Jockey Club Tower, University of Hong Kong, Hong Kong Special Administrative Region; c Department of Geography, National University of Singapore, 1 Arts Link, Kent Ridge, Singapore 117570, Singapore

**Keywords:** Parental migration, Transnational household, Carer, Mental health, South-East Asia, Family practices, Gender, Geopolitical context

## Abstract

The international migration of parents from the global south raises questions about the health impacts of family separation on those who stay behind. This paper uses data collected in 2008 and 2009 for a project on Child Health and Migrant Parents in South-East Asia (CHAMPSEA) to address a largely neglected research area by investigating the mental health of those who stay behind in Indonesia, Philippines and Vietnam to care for the children of overseas migrants. A mixed-methods research design is employed to answer two questions. First, whether carers in transnational (migrant) households are more likely to suffer mental health problems than those in non-migrant households; and secondly, whether transnational family practices and characteristics of migration are associated with mental health outcomes for stay-behind carers. The Self-Reporting Questionnaire (SRQ-20) was completed by carers in selected communities (*N* = 3026) and used to identify likely cases of common mental disorders (CMD). Multivariate logistic regression and thematic analysis of qualitative interviews (*N* = 149) reveal a nuanced picture. All stay-behind carers in the Indonesian sample are more likely than carers in non-migrant households to suffer CMD. Across the three study countries, however, it is stay-behind mothers with husbands working overseas who are most likely to experience poor mental health. Moreover, infrequent contact with the migrant, not receiving remittances and migrant destinations in the Middle East are all positively associated with carer CMD, whereas greater educational attainment and greater wealth are protective factors. These findings add new evidence on the ‘costs’ of international labour migration and point to the role of gendered expectations and wider geopolitical structures. Governments and international policy makers need to intervene to encourage transnational family practices that are less detrimental to the mental health of those who stay behind to care for the next generation.

## Highlights

• Unique focus on mental health of carers in S.E. Asian transnational households.
• Employs innovative integrated mixed-methods research design.
• Finds mothers with husbands working overseas are vulnerable to mental disorders.
• Reveals importance of family practices and geo-political context for carer mental health.

## 1 Introduction and background

The international migration of parents from the global south raises questions about the costs, as well as the benefits, of a livelihood strategy that results in families being divided across national borders. When parents migrate, new ‘transnational’ household arrangements and family practices emerge as childcare is (re)configured in ways that affect the well-being not only of children but also of their carers. This paper addresses a largely neglected area of research by investigating the mental health of mothers, fathers and other family members who stay behind in South-East Asia to care for the children of overseas migrants. Using data collected by our research team in three countries, the analysis examines the impact of parental absence on the mental health of carers in relation to, first, household arrangements, and second, transnational family practices and the characteristics of migration.

Several previous studies of caregiving in the Asia–Pacific region have investigated the health and well-being of family members who provide care, especially for older relatives, but not in the context of migration. Gender differentiation in well-being has emerged as a cause for concern, with female carers at greater risk of poor psychosocial health compared to male carers (Chiou et al., 2005; Ho et al., 2009). Moreover, maternal depression has been found to impact negatively on infant growth in Asia but not in Africa or South America (Stewart, 2007). Such regional differences may reflect variations in poverty and healthcare provision but are also likely to be influenced by different socio-cultural narratives that frame the role of ‘woman and mother’, and Asian motherhood may be especially disempowering (Harpham et al., 2005). However, none of these studies considered carer mental health from the perspective of the transnational family.

Within the substantial body of literature devoted to the study of international migration from Asia, those studies that do consider psychosocial health have largely focused on migrants rather than on those who stay behind. Recent work has examined the well-being of immigrant Asian Americans (Qin, 2008), the difficulties facing migrants who are themselves care workers (Ohno, 2012), the mental health needs of Asian refugees (Hsu et al., 2004), and caregiving in diasporic communities (Lee, 1999; Yoon, 2005). Exceptions which give attention to the impact of adult children's international migration on the health of older parents who stay behind include Kuhn's (2006) work on Matlab, Bangladesh where high rates of out-migration (internal and international) and remittance receipt were found to have a favourable effect on parents' physical health.

There is also a small but growing body of literature that looks at the mental health of children who stay behind in Asia when parents migrate overseas (Battistella and Conaco, 1998; Graham and Jordan, 2011; Hewage et al., 2011; Senaratna et al., 2011), or within China (Fan et al., 2010; Zhao et al., 2014). Nevertheless, there remains a need to extend this work to other non-migrant members of transnational families, especially those who care for the children of migrants (Mazzucato and Schans, 2011).

The few studies that have considered caregiving in South-East Asia in the context of migration have mostly examined the impacts of internal migration on family members who stay behind. Research has reported negative impacts on the intellectual development of Thai children raised by grandparents (Nanthamongkolchai et al., 2011), and several health risks for adults who stay behind in Indonesia, including a greater likelihood of psychosocial distress (Lu, 2012). To the best of our knowledge, the present study is the first to investigate mental health among those who stay behind in South-East Asia to care for children of international labour migrants.

Previous studies point to a number of competing expectations in relation to psychosocial outcomes for stay-behind carers. While the wealth generated by remittances might be expected to mitigate or even eliminate any negative effects of parental absence, the dominance of traditional gender roles in the region (ascribing domestic tasks to women and breadwinning to men) suggests that fathers who stay behind to care for their children while their wives work overseas may face the biggest challenges to their (masculine) identities (Pingol, 2001; Hoang and Yeoh, 2011), which could be detrimental to their mental health. On the other hand, women in low income countries are around three times more likely than men to suffer CMD (Patel, 2001), which suggests that mothers are more at risk of poor mental health than fathers. Further, those mothers coping without the co-resident support of their migrant husbands may be more vulnerable than mothers in non-migrant families. The carer's age and relationship to the child(ren) in their care may also influence their psychological well-being. Grandparent carers may be especially vulnerable to anxiety and stress if they struggle to cope with the physical demands of childcare, although any negative consequences could be outweighed by material security and improved self-esteem associated with the recognition of the importance of their role (Knodel and Chayovan, 2009). The mental health of stay-behind carers merits further examination.

Our aim is to explore the relationships between different family arrangements (non-migrant and transnational) and the mental health of those principally responsible for childcare in selected communities in three countries: Indonesia, Philippines and Vietnam. We adopt a mixed-methods approach to investigate two specific questions: (1) *Are those who stay behind to care for the children of overseas migrants more likely to suffer mental health problems compared to carers in non-migrant families?*; (2) *Are transnational family practices and the characteristics of migration associated with mental health outcomes for stay-behind carers?* The questions are addressed in three stages. To answer the first question, we conduct quantitative analyses that distinguish the main correlates of poor mental health among carers in non-migrant and transnational households within each study country. Next, we examine qualitative data from in-depth interviews with a subsample of these carers in transnational households to ascertain their major concerns and thus possible sources of stress for those who stay behind. Lastly, we combine measures that capture these concerns along with the main correlates identified in the first-stage models to provide a further quantitative analysis predicting poor mental health among stay-behind carers. Qualitative evidence is also used to interpret the quantitative results. This provides a synthesis of the quantitative and qualitative findings and allows us to address the second research question.

## 2 Transnational households in South-East Asia

In the twenty-first century, the global pattern of demand for migrant workers from South-East Asia has shifted away from North America and Europe towards the Middle East and wealthier Asian countries. Crucially, these ‘new’ host countries retain strict controls on migrant labour, ensuring that migrants return to their countries of origin at the end of their contracts. The migrants in our study have all crossed national borders to work in other countries, including Saudi Arabia, Japan, Taiwan and Singapore. Their migration and employment are stringently controlled by legal frameworks that typically allow entry on the basis of temporary 2–3 year work contracts (Piper, 2004). This has three important consequences. First, where low-skilled labour migration is concerned, there is no legal provision for family reunification in destination countries, and taking children (or other family members) with them is very rarely an option for migrant parents. Second, the enforcement of temporary contracts often dictates the frequency of visits home. For both contractual and financial reasons, migrants may wait until the end of a contract before leaving the destination country. Third, temporary contracts are often renewed, or alternative contracts entered into either at the same or a different destination. This process can lead to some migrants spending a decade or more separated from other members of their family. It also introduces an element of uncertainty over when the migrant will finally return. Coping with this uncertainty is just one aspect of contemporary labour migration that may impact negatively on both migrants and those who stay behind.

Those who care for the children of migrants must (re)negotiate ‘care’ within a web of caring relationships among three main participants: the left-behind child, migrant parent(s), and co-resident carer(s) (Graham et al., 2012). Family practices, including contact with migrant parents, can therefore be expected to influence both the quality of childcare and the well-being of carers. Family members are separated geographically, but generally over far greater distances than the urban-rural migrants discussed by Knodel and Saengtienchai (2007), and certainly with greater barriers to communication or visits. Thus patterns of contact between carers and migrant parents are not only a reflection of personal relationships but will also be structured by the geopolitical contexts in which migration occurs and the gendered norms around responsibility and ‘care’ (Parreñas, 2005).

## 3 Mixed-methods research design

The study examines multiple influences on the well-being of those caring for young children by using the complementary strengths of qualitative and quantitative methods to analyse and interpret data collected for a project investigating Child Health and Migrant Parents in South-East Asia (CHAMPSEA). In 2008, we conducted structured surveys with several members of both transnational and non-migrant households in non-metropolitan, high-outmigration areas in four countries – Indonesia, Philippines, Thailand and Vietnam. Transnational households were defined as households in which one or both parents had been working abroad and not returned home in at least the six months prior to interview. The definition of non-migrant households required both parents to have been usually resident at the same address for at least the previous six months. CHAMPSEA study sites were first identified by in-country experts as provinces with international out-migration rates that are higher than their respective national average. These were East and West Java in Indonesia, Laguna and Bulacan in the Philippines, and Thai Binh and Hai Dong in Vietnam. After identifying non-metropolitan communities with high out-migration rates within each province, we then purposely selected communities to reflect a variety of contexts such as their migration history (long-established or more recent) or urbanization in the case of the Philippines (rural or more urbanized communities in provinces with long-established history of international out-migration) (Graham and Yeoh, 2013). In the absence of national data on migrant parents, we used a replicable sampling strategy adapted from sentinel site surveillance methods that involved screening households in selected communities. Households with a child in one of two age groups (3–5 years, or 9–11 years), and with two parents who were either both co-resident (non-migrant households) or where one or both were international migrants (transnational households), were recruited to the study according to pre-set quotas. Divorced and single-parent households were not included, and only one index child was recruited in each qualifying household (see Graham and Yeoh, 2013). Households identified the index child's principal caregiver, who was then interviewed. The same strategy was used in all study countries to provide comparable datasets, which are of sufficient size for the current study. The country samples are not nationally representative but, for simplicity, we use the country name when referring to the data. Ethical approval for the study was granted by the University of St Andrews (UK), National University of Singapore, Center for Population and Policy Studies, Gadjah Mada University (Indonesia), Scalabrini Migration Center (Philippines), and the Asia–Pacific Economic Center (Vietnam).

The survey instruments included measures of carer mental health and we distinguish three groups of carers within transnational households, differentiated by their gender and relationship to the index child (mother-carer/father-migrant; father-carer/mother-migrant; other-carer/parent(s)-migrant). The last of these groups includes a variety of different carers (grandmothers, grandfathers, aunts, uncles and older siblings) and households where one or both parents were international migrants. We combined these for the current analysis due to the small numbers in each subgroup and we therefore focus especially on parent carers. We also collected data on the mental health of carers in non-migrant families, nearly 90 percent of whom are mothers (90.8% in Indonesia, 92.6% in Philippines, 82.4% in Vietnam). We treat carers in non-migrant households as a single group because even working mothers are involved in the daily care of their children. This allows us to compare the mental health of different carers in transnational households with the mental health of carers in non-migrant households. Our geographical focus is on three of the CHAMPSEA study countries. Thailand is excluded because very few fathers (*n* = 3) stayed behind to look after their children in the Thai communities surveyed, limiting gender comparisons. As we expect gender disparities to be important (Yee and Schultz, 2000), the current study uses data for Indonesia, Philippines and Vietnam. A few cases are dropped in each country (9 for Indonesia, 10 for Philippines and 1 for Vietnam) where data are missing, reducing the overall sample by less than 1 percent and providing a total analytical sample of 3026 cases. Table 1 shows the sample composition by country and household arrangements, as well as providing descriptive statistics for all other model variables.

The descriptive statistics reveal some important differences between the samples for the three study countries. For the Philippines and Vietnam, for example, around a fifth of carers have primary education or less, whereas over half of Indonesian carers have low levels of education. Further, over 70 percent of Filipino carers have at least upper secondary education, compared with less than a fifth in the Indonesian and Vietnamese samples. While a high proportion of carers reported having someone to turn to if they had a problem, this is lowest in the Indonesian sample at 87 percent. The mean age of carers is around 38 years old and is similar across the country samples, although it is 2 years higher among carers in transnational households. The asset-based measure of relative household wealth classifies households by wealth quintile (low = first and second; medium = third and fourth; high = fifth) within each country sample for the country-specific models and across the pooled samples for the three study countries for the combined transnational sample. Thus it can be seen from Table 1 that transnational households tend to be relatively wealthier, as a higher proportion of households in the combined sample are in the high wealth category. Nearly 50 percent of migrant parents were working in other Asian countries (especially Taiwan, Malaysia and Hong Kong), 38 percent in Middle Eastern countries (mainly Saudi Arabia, United Arab Emirates and Qatar) and only 14 percent in other countries around the world. The majority of transnational households (84 percent) had received remittances in the previous six months and were in at least weekly contact with migrant parents (66 percent). However, over a third of carers reported infrequent contact with absent parents, which may be associated with poor mental health.

The analysis is reported sequentially in three stages, although in practice an iterative process was employed, with the last two elements informing each other. First, logistic regression models predicting carer CMD were fitted to survey data from non-migrant and transnational households in each of the study countries – Indonesia (*n* = 1025), Philippines (*n* = 990) and Vietnam (*n* = 1011) to determine whether stay-behind carers are more likely to suffer poor mental health and to identify significant correlates of carer CMD within the country samples.

Poor mental health is assessed by the Self-Reporting Questionnaire (SRQ-20), a screening tool for psychiatric disturbance recommended for use in developing countries and now validated in many cultural contexts (Tuan et al., 2004). All carers of children were asked 20 yes/no questions relating to symptoms of depression and anxiety in the previous 30 days. These cover common problems associated with emotional distress, such as headaches, poor appetite, nervousness, difficulty in making decisions, suicidal thoughts and tiredness. Scores added the number of ‘yes’ answers and, following De Silva et al. (2007) work in Vietnam, we selected a commonly used cut-off, with scores of 8 or more denoting probable cases of common mental disorders (CMD). On this measure, the prevalence of CMD is similar for the Philippines and Vietnam, at 14.3 percent and 16.1 percent respectively, but considerably higher for the Indonesian sample at 26.8 percent. Prevalence among carers in transnational households is 20.6 percent (see Table 1).

Next, we undertook a thematic analysis of qualitative interviews, conducted in 2009 with a subsample of carers from transnational households, to reveal the dynamics of family practices articulated by carers themselves. The subsample was drawn separately for each country by using a 16-cell matrix constructed from the survey data and based on the carer's relationship to the index child, and the index child's physical and psychological health. Matrices were divided on the *x*-axis into four categories according to whether the carer was the child's (i) mother, (ii) father, (iii) close female relative or (iv) other. The *y*-axis was also divided into four categories according to combinations of the physical health (underweight measured by weight-by-age z scores) and psychological well-being (measured by the Total Difficulties Score from the Strengths and Difficulties Questionnaire (SDQ), with scores of 17 and above denoting psychological difficulties (Goodman, 1997)) of the child being cared for: (i) good physical and psychological health, (ii) good physical but poor psychological health, (iii) poor physical but good psychological health, and (iv) poor physical and psychological health. The samples from each of the study countries were divided across eight such matrices according to the index child's age (3–5 or 9–11 years) and the household's migration status (non-migrant, father migrant, mother migrant or both parents migrant). Quotas of 1–3 interviews from households in each of the populated cells were set according to the number of households in the cell, thus ensuring a spread of carer interviews across households of different types and circumstances (52 in Indonesia, 48 in Philippines, and 49 in Vietnam). In the present study SRQ scores from the main survey are used to select likely cases of poor mental health among carers from transnational households who participated in the interviews. Interviewing carers in local languages enabled us to capture their own understandings and experiences of living in a transnational household. Some carers were stay-behind mothers whose husbands were working overseas; some were fathers who have adapted to a new role as childcare giver in the absence of their migrant wives; yet others were grandparents (usually grandmothers), aunts, uncles or older siblings who had assumed the role of principal caregiver on the migration of one or both of the index child's parents. All transcripts were translated into English by in-country researchers who also acted as interviewers, and pseudonyms are used to ensure anonymity. The translated transcripts were then coded and analysed using NVivo software.

In the final stage of the analysis, we use the insights from the qualitative interviews, along with the results of the first-stage models, to design a further set of logistic regression models predicting CMD among carers in transnational households only (*n* = 1576). The geopolitical context of migration is investigated by including the migrant destination, as well as measures capturing dimensions of family practice - remittance receipt, contact with migrant parent, and working outside the home - raised as concerns by carers themselves. A test revealed no problems of multicollinearity; these measures are therefore included individually in the final models. Carer age and gender are also entered as independent variables. Our purpose is to investigate the ways in which wider social and political structures combine with family practices and correlates of CMD to influence the mental health of carers in transnational households. We therefore pool the data for the three study countries to highlight commonalities across the combined transnational sample.

## 4 Correlates of poor carer mental health in three countries

The lack of previous comparable studies means that the analyses are inevitably exploratory. The country-specific multivariate models presented below explore the mental health of carers in different types of household arrangement and address our first research question. A range of additional variables is included to account for the duration of parental absence, household and carer characteristics and whether the carer receives social support. Among these are two variables indicating whether the carer, and/or other member of the household has a (physical) disability^1^. We include these variables despite small numbers because they could be important confounders of the relationship between parental migration and carer mental health. A sensitivity test (not shown) revealed the stability of the overall modelling results when the carer disability variable is excluded from the country-specific models.

**Fig. 1:**
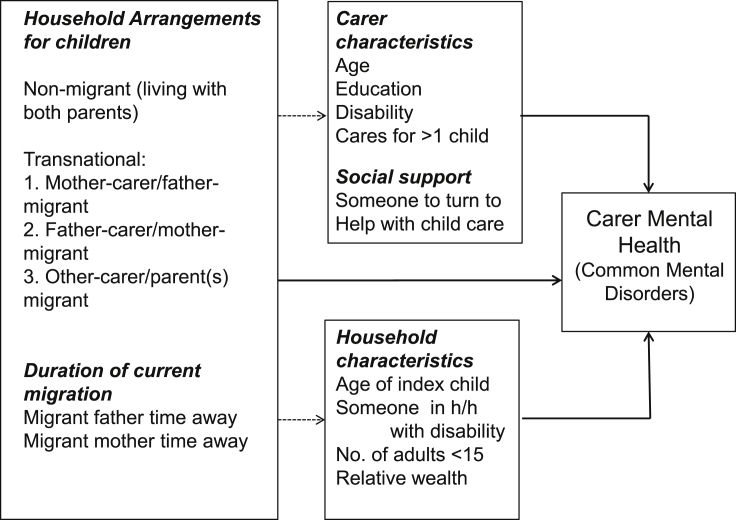
Analytical framework and model variables.

Fig. 1 provides a summary of the analytical framework for the first-stage analysis and a full list of variables. The same model is fitted for each of the study countries separately, with the aim of identifying major correlates of CMD and determining whether carers in transnational households are at greater risk.

Table 2 reports the results from the models and identifies several significant correlates of CMD among those caring for children in each of the three countries. It is apparent that the number of correlates identified for the Indonesian sample is greater than for either the Philippines or Vietnam, although some factors are common across two or more country samples. For example, low educational attainment (primary schooling or less) is associated with poorer outcomes for carers in Indonesia and Vietnam, but not in the Philippines, while carer disability is a significant correlate of CMD among carers in Indonesia and Philippines, but not in Vietnam. In the latter case, the wide confidence intervals for Indonesian and Vietnamese carers reflect the small numbers involved and no firm conclusions can be drawn. However, Filipino carers who have a (physical) disability are over two and a half times more likely to suffer CMD than those who do not. Further, the presence of someone with a disability in the household is a correlate only for Filipino carers, significantly increasing the odds of CMD. In Vietnam it is those looking after children aged 9–11 years who have higher odds of CMD compared to those caring for younger children. Interestingly, carers looking after more than one child, who comprise over 60 percent of all carers, are no more likely to suffer mental health problems than carers of single children in any of the countries. Other correlates of CMD among carers are related to the absence of social support (not having someone to turn to in Indonesia, and not having help with childcare in Vietnam), and poverty (low household wealth in all three countries).

All the correlates above are independently associated with mental health outcomes among carers whether they live in transnational or non-migrant households. Some are related to household arrangements, but a check revealed no problems of collinearity. For example, the receipt of remittances from overseas earnings results in transnational households being, on average, wealthier than non-migrant households; however the overall correlation is low, in part because not all migrants send remittances. Accounting for all these factors in models that include household arrangements provides a robust test of mental health outcomes for carers in transnational households, differentiated by who provides childcare and whether the mother and/or father works overseas, compared to outcomes for carers in non-migrant households. The models also account for the time the mother and/or father has been away, measured as the proportion of the index child's life during which the parent has been working overseas. The results suggest that the duration of parental absence is not a significant correlate of carer CMD.

After controlling for possible confounders, some stay-behind carers in transnational households remain significantly more likely to suffer poor mental health compared to carers in non-migrant households in the same communities. For stay-behind Indonesian carers, whether mothers with migrant husbands, fathers with migrant wives, or other carers looking after children while parents work overseas, the odds of CMD are around twice those for carers in non-migrant households. The same relationship is not apparent for stay-behind father-carers in the Philippines and Vietnam, nor for other (non-parental) carers in Vietnam, whereas other carers in the Philippines have the highest odds of CMD compared to carers in non-migrant households (OR = 2.45, *p* < 0.05)^2^. The lack of a significant association between Filipino father-carers and poor mental health may be questioned because of the relatively low number of cases in this group. However, it could also be that Filipino fathers adjust to new caring roles more easily than Indonesian fathers or that, as studies of gender difference in mental health suggest, they and their Vietnamese counterparts report fewer symptoms. The most consistent finding across all three country samples is the higher likelihood of CMD among mothers caring for children while their husbands work overseas. The association is especially apparent for stay-behind Vietnamese mother-carers who are over two and a half times more likely to suffer CMD than carers in non-migrant households. Evidence from the qualitative interviews is now considered, to identify concerns expressed by carers themselves.

## 5 The concerns of carers in transnational households

The last two stages of the analysis investigate the second research question, whether transnational family practices and migration characteristics are associated with mental health outcomes for stay-behind carers. Turning to the qualitative interviews with carers in transnational households, we identified three themes – contact with migrant parent, remittance receipt and working outside the home – that summarise sources of stress for some respondents and therefore may have negatively impacted their mental health. By way of illustration, we draw here on two interviews with stay-behind mothers that reflect common concerns, including those raised by stay-behind fathers and other caregivers.

Our previous research revealed a deficit in children's subjective well-being when communication with migrant parents is not maintained (Graham et al., 2012) and the importance of communication for good parent–child relations in transnational families has recently been highlighted by Haagsman and Mazzucato (2014). Ethnographic work has also made a valuable contribution to understanding long-distance communication (Parreñas, 2005; Fresnoza-Flot, 2009; Madianou and Miller, 2011). However, the important question of whether contact, or lack of contact, between migrant parents and stay-behind carers might be associated with the mental health of carers has attracted little scholarly attention. The narrative of an Indonesian mother-carer provides insight into this neglected dimension of the ‘care triangle’.

Mitin, a 40-year-old Indonesian mother of three children whose husband had left to seek work in Malaysia some four years before, was visibly upset – alternating between almost breaking down in tears and laughing bitterly – when speaking of her husband with whom she had had no contact for the last eight months. Mitin's husband, who had first left for Malaysia “to look for money so that we'd be able to send our children to school” was able to send some money home during the first few years but on his return some eight months ago during the *Idul Fitri* (celebrations at the end of the Muslim fasting month), Mitin discovered that all was not well:

He brought no money [home] at all. Well, just enough to buy a roundtrip ticket [to return to Malaysia]. All his salary ran out to manage his work permit [possibly meaning that her husband had to pay for a fake permit]. Then he left again …. It's been difficult for me since he left without giving me any money, no money at all.

Apart from one phone call to report his safe arrival in Malaysia, Mitin's husband had not been in contact:

[ … ] it's been almost 8 months … and it'll be *Idul Fitri* again … but he hasn't sent any news. He is supposed to know that his children [need money] for school … and their daily needs [ … ] and he hasn't even sent any news.

To support her family through this difficult time when her husband has sent neither word nor money, Mitin had quickly sprung into action

But, *Alhamdulilah* [“thank God”], I can fulfil this family's needs … I can find money to send my children to school [… ] I set up a small stall to sell *tempe* to provide for the children's daily needs, [ … ] I work at the rice fields and use my wages to buy soybean.

While Mitin apparently drew strength by recalling her mother's advice “when you're married, don't be a burden to your husband, don't rely on your husband, you have to be an independent woman”, she also confessed that her health had been affected:

Since their father left, I often get a headache [ … ] I feel dizzy, [as though] the world is turning around [ … ] maybe I'm too tired and I do not sleep enough [ … ] I work every day for my children's pocket money, for us to eat [ … ] I have a lot of things to think about [ … ] and nobody helps me.

As Mitin's case indicates, absence without contact – especially one that is prolonged without reason – creates a major breach in the transnational family and has a deleterious effect on the psychological well-being of the stay-behind carer. As scholars have noted, phone calls (or other communications technologies) function as the “social glue of migrant transnationalism” (Vertovec, 2004: 219) and stay-behind family members are likely to experience frustration and anxiety over the failure to communicate effectively across transnational spaces (Carling, 2008).

The regularity with which remittances are received from their husbands also influences the stress levels women face in enacting their roles as ‘good mothers’. Mitin's anxieties relate to *both* her husband's lack of contact and his failure to provide the family with financial support, which in turn required her to seek work outside the home. In this respect, Mitin's husband was failing in his role as breadwinner for his family.

That the dominance of traditional gender roles continues to frame women's (and men's) self-expectations is also apparent in our interview with Phi Yen, a 34-year-old Vietnamese mother of two whose husband had left nine months before for forestry work in Brunei. As her husband had not been able to send any money home as yet, and the bank loan undertaken to finance his migration generated interest of “nearly 300,000 dong each month”, Phi Yen took on farm work during the harvest months to make ends meet despite poor physical health (she complained of dizzy spells, “heart problems” and “wear and tear”). She is proudest of herself for being able to “do everything for the family”. Conversely, her main source of distress seems to stem from having to do men's work:

I do feel pity for myself [… ] I have to do heavy work such as carrying padi [sacks] or other heavy work that should be done by a man. But I have to motivate myself that the hard work is for the children.

Stay-behind mothers whose husbands are working overseas comprised the group identified in the first-stage models as consistently likely to suffer poor mental health across the three country samples. Their stories provide insight into sources of stress and anxiety in their daily lives but are also bound up with wider gendered narratives and geopolitical contexts. Phi Yen felt pity for herself because she had to do work that “should be done by men” to provide for her children, and Mitin struggled with dual roles as breadwinner and carer when her husband stopped sending money home. The burden of extra duties weighs heavily on Phi Yen and Mitin and is probably detrimental to their physical, as well as psychological, health. However, it is the lack of contact and remittance receipts that are the main causes of concern. Although we do not have the husbands' accounts of their failure to conform to their pre-scripted role, it appears that both men have been caught up in debt-financed migration.

## 6 The role of transnational family practices and migration characteristics

The two women's stories, while each unique, illustrate concerns shared by other stay-behind carers. Uncertainties about contact with migrant parents were not uncommon and the stresses of depending upon the receipt of remittances to sustain the family were expressed by many. When remittances were not sent, stay-behind carers often spoke about having to earn money by working outside the home. These common themes inform the next series of hierarchical logistic regression models predicting CMD among carers in transnational households. Data for the three study countries are pooled to examine the associations between carer CMD and remittance receipt, contact with migrant, and working outside the home, while accounting for all the significant correlates previously identified. Migrant destination is also included in these models.

The first model in Table 3 (Model A) reports the relationships between carer CMD and migration characteristics and transnational family practices. Carer's age and gender are included to account for differences in reported health between females and males, as well as differences between generations (parents and grandparents). Results suggest that female carers are more likely to suffer CMD than male carers, although the association only approaches significance, while age is not related to poor mental health. However, three of the other variables are significantly associated with CMD. Carers who have infrequent contact with the migrant parent are almost twice as likely to experience CMD as those who are in weekly contact. This suggests that communication from a migrant parent makes a significant contribution to the carer's well-being. In addition, financial support appears to be important, as the odds of CMD for carers who have received remittances from migrant parents in the last six months are over 30 percent lower than for those who have not. For migrants working overseas in low-paid jobs, not only is saving enough of their earnings to send regular remittances a financial challenge but the costs of phoning home combined with the conditions of their employment also serve to limit their contact with family members who stay behind. Some domestic workers, for example, have their mobile phones removed by employers and their opportunity for contact is severely curtailed by having little free time during the average week (Human Rights Watch, 2012). Such structural constraints may vary depending on the migrant's destination and our findings indicate that migrants in the Middle Eastern face particular difficulties, as the likelihood of CMD among carers in households with a migrant parent in the Middle East is considerably higher than for carers of children whose mother and/or father works in other Asian countries (OR = 1.83, *p* < 0.001). Contrary to expectations, working outside the home is not significantly associated with the likelihood of CMD for carers in transnational households.

The second model (Table 3, Model B) adds all but one of the other correlates of carer CMD identified in the first-stage country-specific models (Table 3, grey panel). The exception is household wealth, which is included in the final model. Migrant parents, especially perhaps migrant mothers, often engage in active parenting from afar, or what Madianou and Miller (2011) term ‘mobile phone parenting’. However, the frequency of contact may be influenced by other factors such as carer education, which was identified earlier as a correlate of CMD among carers in Indonesia and Vietnam. The results confirm an attenuation of the positive association between infrequent contact and CMD, but the relationship remains significant. Educational attainment also retains an independent effect; the odds of CMD for carers with at least higher secondary qualifications are less than half those for carers with primary school education or less. Receiving remittances in the last six months is also protective for CMD, whereas stay-behind carers of children whose mother and/or father is working in the Middle East are, as in Model A, significantly more likely than other stay-behind carers to experience CMD.

In the final model (Table 3, Model C) we add household wealth and a variable for the study country that accounts for unobserved differences between the three countries. Modern communications can be relatively costly, and we therefore expect household wealth to influence the frequency of contact between migrants and carers who stay behind. Our findings confirm our expectation. As Model C shows, the inclusion of household wealth attenuates the higher odds of CMD for carers who have infrequent contact with migrant parents, suggesting that variations in contact frequency are associated with relative household wealth. The qualitative interviews provide further evidence of this relationship and how it can change over time. As a Vietnamese father who cared for his child while his wife worked overseas remarked, “Before, it was a bit tighter for us and she called home once a month only. But now she calls home once a week or even twice or 3 times a week if there are any urgent issues.”

Model C also demonstrates that wealthier households are more likely to receive remittances, as the association between remittance receipt and carer CMD is no longer significant once household wealth is included in the model. Further, carers in both medium and high wealth transnational households are significantly less likely to experience CMD compared to carers in low wealth transnational households. This finding is consistent with other studies which have shown an association between poverty and CMD risk in developing countries (Patel and Kleinman, 2003) but also suggests that the wealth of transnational households depends on the regular receipt of remittances.

After accounting for all other factors, we find a significant relationship between carer gender and CMD, with stay-behind female carers more likely than male carers to report symptoms of poor mental health. Most interestingly, controlling for wealth and study country increases the higher odds of CMD for carers in households where a parent is working in the Middle East compared to those where a migrant parent is working in another Asian country (OR = 2.02, *p* < 0.001). It should be noted, however, that these results are associated with the much higher numbers of migrant parents from Indonesia and the Philippines who are working in the Middle East, compared to numbers in the Vietnamese sample. Further, there remain significant differences between the Philippines and the other two study countries. The odds of CMD are higher for both Indonesian (OR = 2.41, *p* < 0.001) and Vietnamese (OR = 1.88, *p* < 0.05) carers. The exploitative nature of the migration industry in Vietnam has resulted in greater levels of debt among Vietnamese transnational households in our study communities (Hoang and Yeoh, 2012) and this may be contributing to poorer mental health among Vietnamese carers. It is also possible that more migrant parents from both Vietnam and Indonesia are working in manual or unskilled jobs compared to Filipino migrants, and are therefore able to contribute less to household finances. The employment conditions of such migrants likely reflect the imbalance of power between the sending and host nations, which can influence transnational family lives. Family practices, including migrant parents maintaining contact with and sending remittances to those who stay behind, are thus subject to multiple influences and it is these practices which, our findings suggest, impact on the psychosocial well-being of carers.

## 7 Conclusions

Previous work on the health consequences of internal migration in Indonesia (Lu, 2012) recognised the need for research on the psychosocial costs of international migration for those who stay behind, as well as more explicit attention to the role of remittances and contact among family members. We have advanced this agenda by investigating the two research questions posed in the introduction using a combination of qualitative and quantitative evidence from the CHAMPSEA project.

First, the comparative analysis of the three study countries shows that *some* carers in transnational households are more likely than carers in non-migrant households to experience mental health problems. Higher odds of CMD were found in all three country samples for mother-carers whose husbands were working overseas, in Indonesia for father-carers whose wives were working overseas, and in Indonesia and the Philippines for other carers in transnational households. The general pattern of disadvantage in the Indonesian sample was not replicated for the Philippines or Vietnam, where CMD prevalence rates were lower. Among possible confounders, only the association between CMD and low household wealth was common to all three countries. The finding that living in a low-wealth household is associated with a significantly higher likelihood of CMD among carers, while stay-behind mother-carers in the three study countries also have higher odds of CMD, indicates that the benefits of relative financial security do not always outweigh the costs of transnational family life. For mother-carers in low income transnational households there is a double jeopardy. The processes that underlie these costs are unobserved in the country-specific models but we surmise that they are related in part to a complex interplay between gendered expectations of ‘good parenting’ and the reconfigured role of the migrant parent within the transnational family.

The qualitative interviews provide more specific insight into why stay-behind mothers are almost twice as likely to suffer common mental disorders compared to their counterparts in non-migrant households, and more than two and a half times as likely in the Vietnamese sample. When husbands go overseas to work, mothers often have to take on roles (such as agricultural work) previously performed by the men. Not only does this impose a physical burden but it may also be resented if the migrant husband does not fulfill his part of the bargain by regularly sending remittances home. The absence of migrant husbands also removes an important source of social support, which can be protective for depression and anxiety. Further, inevitable changes in the relationship between children and their non-resident father may become a source of stress for the mother if an emotional gap develops as children become used to having only one parent around. This may be particularly so if the father attempts to impose discipline on his children from a distance or during short visits home, as Parreñas (2008) has argued. In addition, prolonged separation may threaten the survival of the marriage, frequently leaving stay-behind mothers facing uncertainty and economic hardship. While stay-behind fathers may encounter similar sources of stress in the absence of their migrant wives, the qualitative evidence suggests that they do so to a lesser degree. For example, many fathers who become the principal carers for their children share the burden with other (female) members of their extended family (Hoang and Yeoh, 2011). The modelling results suggest that only stay-behind father carers in Indonesia have significantly higher odds of CMD and this may be due as much to the employment circumstances of their migrant wives as it is to the need for them to redefine their gender role within the household.

The second research question asked more explicitly about the role of transnational family practices and migration characteristics. The narratives of two stay-behind mothers illustrate how the migration of their husbands had, at times, negatively impacted on their ability to care for their children. Remittances and communication from a migrant husband are signs that he is fulfilling his roles as father and spouse. Migrants who fail to communicate or send remittances may be seen as betraying expectations, and stay-behind carers may have to seek employment outside the home to provide for the children in their care. These circumstances are likely to increase CMD among carers by causing stress and uncertainty. The failure to communicate or send remittances regularly is not always a matter of choice, and destination-specific barriers deserve greater recognition. The modelling results (Table 3) indicate that both transnational family practices (contact and remittance receipt) and migration characteristics (migrant destination and sending country) influence the likelihood of CMD for stay-behind carers. Infrequent contact with the migrant, not receiving remittances in the past six months and caring for children in a transnational household where the migrant is working in the Middle East are all associated with higher odds of carers experiencing CMD. In addition, stay-behind carers in Indonesia are most likely to suffer mental health problems. This answers our second research question, but also raises further questions.

While the effects of family practices are modified by household wealth, the mental health deficit for carers of children whose parents are working in the Middle East is not. The qualitative evidence suggests that concern about the welfare of the migrant parent is a possible source of stress for the carer, as stories of migrants being suddenly sent home must surely circulate within communities. However, more research is needed to determine why the odds of CMD are significantly higher for these carers. Future work will investigate the possible processes underlying this finding, including whether it is related to particular destinations within the region and/or particular types of migrant employment. Equally, the significantly higher likelihood of CMD among stay-behind carers in the Indonesian sample, whatever their gender, wealth or personal circumstances, requires further investigation. The interviews with carers lead us to suspect that both are indicative of international circuits of labour mobility structured by geopolitical contexts that severely constrain family practices in ways detrimental to psychosocial well-being.

This study has demonstrated that living in a transnational household and caring for children left behind is associated with an increased likelihood of poor mental health for carers in particular circumstances. The quantitative analyses distinguished major correlates of carer CMD and investigated differences among carers in transnational households, while the thematic analysis provided greater insight into the processes at work. Despite utilising the strengths of both approaches, the study is subject to limitations arising from the nature of the main survey. First, the use of cross-sectional data means that the direction of causation cannot be ascertained. Further, while individual circumstances can and do change over time, this dynamic is not captured in the quantitative analyses. Detailed longitudinal data, currently not available, would be needed to explore this further. Secondly, given the sampling strategy, the study findings cannot be generalised beyond the study communities. It is possible, for example, that stay-behind carers in major metropolitan areas in the study countries have different mental health outcomes. Nevertheless, by identifying significant differences in the likelihood of CMD among carers in transnational and non-migrant households and by establishing a connection between poor mental health and transnational family practices, we have fulfilled our aim of demonstrating, for the first time, important vulnerabilities among those providing childcare in transnational households in South-East Asia. These deserve much more research attention. The agenda for future work should not only include further studies on transnational households in other communities but also attend to the ways in which geopolitical context and gendered expectations play a role in creating vulnerabilities to poor mental health.

The viability of transnationalism as a way of “doing family” depends to a considerable extent on geographically separate family members playing their part, often in accordance with gender role expectations. The migration of a parent – whether a father or a mother – is part of a livelihood strategy that seeks to secure a better future, especially for children. However, transnationalism involves often hidden costs as well as potential benefits. For some carers who stay behind, the promise of financial security comes at a cost to their mental health. In less developed parts of South-East Asia such as the sites where the CHAMPSEA project was conducted, the absence of remittances has severe implications for the well-being of non-migrant family members, including stay-behind carers, who are heavily dependent on this source of income for daily subsistence and debt repayment, as well as peace of mind that their sacrifice has been worthwhile. The findings of this study suggest that the difficulties faced by many of these families may be shaped by geopolitical structures over which they have no influence, and which need to be addressed by governments and international policy-makers to encourage transnational family practices that are less detrimental to the mental health of those who stay behind to care for the next generation.

## Figures and Tables

**Table 1:** Descriptive statistics for model variables by country and combined sample of transnational households.

Characteristics				Indonesia	Philippines	Vietnam	Combined sample (transnational households)	
				%	%	%	%	
Carer common mental disorders (CMD)				26.73	14.34	16.12	20.62	

*Household arrangements*								
Non-migrant household				48.78	49.39	45.60		
Mother-carer/father-migrant				16.29	34.75	17.71		
Father-carer/mother-migrant				19.22	5.66	20.18		
Other-carer/Parent(s)-migrant				15.71	10.20	16.52		

*Duration of current migration*								
Migrant father time away (mean months)				*4.40*	*7.44*	*5.43*		
Migrant mother time away (mean months)				*8.00*	*2.67*	*7.60*		

*Carer characteristics*								
Age in years (mean)				*38.19*	*37.21*	*38.02*	*40.46*	
Male							32.04	
Female							67.96	

Primary education or less				61.66	23.54	19.68	*36.99*	
Lower secondary				19.80	4.14	63.01	28.43	
Upper secondary or more				18.54	72.32	17.31	34.58	

Carer disability:			No	92.68	90.91	97.73	91.50	
			Yes	2.83	6.67	0.30	3.36	
			Unknown	4.49	2.42	1.98	5.14	

Cares for >1 child				52.49	67.37	70.92		
Someone to turn to				86.93	95.86	95.25	91.94	
Help with childcare				52.20	41.52	63.2	55.77	
Carer works outside home							46.38	

*Household characteristics*								
Index child aged 9–11				49.76	50.20	50.54	50.32	
Someone in h/h with disability				5.37	13.84	2.87	5.77	
No. of adults >15 years (mean)				*2.29*	*2.55*	*2.34*		

Low wealth				40.10	41.21	40.06	31.41	
Medium wealth				41.37	39.80	40.65	42.77	
High Wealth				18.54	18.99	19.29	25.82	

Contact with migrant:	Once a week						65.99	
	Infrequent						34.01	
	Received remittances in last 6 months						83.63	

Migrant destination: Other Asian country							48.03	
Middle East							38.01	
Other International							13.96	

Country:		Indonesia					33.31	
		Philippines					31.79	
		Vietnam					34.90	

*N*				1025	990	1011	1576	

Numbers in italics are means.

**Table 2:** Logistic regression models predicting CMD among carers of children in (a) Indonesia, (b) Philippines, and (c) Vietnam.

		Indonesia		Philippines		Vietnam	
		*n* = 1025		*n* = 990		*n* = 1011	
		OR	95% CI	OR	95% CI	OR	95% CI
*Household arrangements*							
*Non-migrant household*		*1.00*		*1.00*		*1.00*	
Mother-carer/father-migrant		1.90*	1.12, 3.23	1.80*	1.05, 3.10	2.67**	1.45, 4.90
Father-carer/mother-migrant		2.01*	1.18, 3.42	1.44	0.46, 4.45	0.74	0.38, 1.44
Other-carer/Parent(s)-migrant		1.93*	1.05, 3.55	2.45*	1.01, 5.96	0.81	0.33, 1.97

*Duration of current migration*							
Migrant father time away		1.00	0.98, 1.01	0.99	0.97, 1.01	0.98ˆ	0.96, 1.00
Migrant mother time away		1.00	0.99, 1.02	0.98	0.95, 1.02	1.00	0.98, 1.02

*Carer characteristics*							
Age in years		0.98*	0.97, 1.00	1.01	0.99, 1.03	1.02	0.99, 1.04
*Primary education or less*		*1.00*		*1.00*		*1.00*	
Lower secondary		0.81	0.54, 1.21	0.63	0.23, 1.75	0.65ˆ	0.42, 1.02
Upper secondary or more		0.51**	0.32, 0.84	0.77	0.49, 1.21	0.36**	0.18, 0.71
Carer disability	No	*1.00*		*1.00*		*1.00*	
	Yes	7.58***	3.26,17.62	2.65**	1.45, 4.84	4.48	0.37,54.68
	Unknown	1.37	0.64, 2.94	0.59	0.15, 2.27	0.59	0.15, 2.35
Cares for > 1 child		0.98	0.73, 1.32	0.85	0.57, 1.26	0.87	0.58, 1.30
*Carer social support*							
Someone to turn to		0.65 *	0.43, 0.99	0.47 ˆ	0.22, 1.00	1.96	0.79, 4.87
Help with child care		0.94	0.69, 1.27	0.77	0.51, 1.15	0.62 **	0.43, 0.88

*Household characteristics*							
Index child aged 9–11		0.94	0.69, 1.28	1.32	0.90, 1.95	1.45 *	1.01, 2.09
Someone in h/h with disability		1.31	1.31 0.71, 2.45	1.71 *	1.04, 2.80	1.53	0.61, 3.85
No. of adults >15 years		1.12	0.95, 1.31	1.08	0.94,1.23	0.92	0.76, 1.12

*Low wealth*		*1.00*		*1.00*		*1.00*	
Medium wealth		0.56***	0.40, 0.77	0.56*	0.36, 0.88	0.60*	0.41, 0.89
High Wealth		0.47**	0.29, 0.76	0.45**	0.25, 0.82	0.72	0.43, 1.20
Log likelihood			−553.5793		−382.0267		−420.8320

OR = odds ratio; ***p < 0.001, **p < 0.01, *p < 0.05, ˆ*p* < 0.1.

**Table 3:** Logistic regression models predicting CMD among carers of children in transnational households (combined sample).

	*n* = 1576					
	Model A		Model B		Model C	
	*OR*	95% CI	OR	95% CI	OR	95% CI
*Carer characteristics*						
*Male*	*1.00*		*1.00*		*1.00*	
Female	1.30ˆ	0.97, 1.75	1.35ˆ	0.99,1.85	1.42*	1.04, 1.96

Age in years	0.99	0.98, 1.00	0.98**	0.97, 1.00	0.99*	0.98, 1,00

*Contact with migrant*						
*Once a week or more*	*1.00*		*1.00*		*1.00*	
Infrequent	1.94***	1.48, 2.54	1.66***	1.26, 2.21	1.34ˆ	1.00, 1.81

*Remittances in last six months*						
*No*	*1.00*		*1.00*		*1.00*	
Yes	0.63**	0.45, 0.87	0.66*	0.47, 0.92	0.77	0.55, 1.08

*Migrant destination*						
*Other Asia*	*1.00*		*1.00*		*1.00*	
Middle East	1.83***	1.34, 2.38	1.86***	1.39, 2.49	2.02***	1.44, 2.83
Other international	0.72	0.46, 1.12	0.84	0.53, 1.32	1.03	0.64, 1.63

Carer works outside home	1.02	0.78, 1.33	0.98	0.75, 1.30	0.93	0.70, 1.22

Carer education						
Primary or less			*1.00*		*1.00*	
Lower secondary			0.79	0.56, 1.12	0.84	0.58, 1.23
*Upper secondary or mor*e			0.40***	0.28, 0.57	0.61*	0.41, 0.92

Carer disability						
No			*1.00*		*1.00*	
Yes			2.78**	1.50, 5.16	3.36***	1.77, 6.38
Unknown			0.87	0.49, 1.53	0.86	0.49, 1.54

Social support						
Someone to turn to			0.82	0.53, 1.27	0.87	0.55. 1.35
Help with childcare			0.94	0.72, 1.22	0.91	0.70, 1.19

Household characteristics						
Index child aged 9–11			1.15	0.89, 1.49	1.21	0.93, 1.58
Someone in h/h has disability			1.75*	1.06, 2.89	2.06**	1.23, 3.46
Low wealth					*1.00*	
Medium wealth					0.64**	0.48, 0.87
High wealth					0.59**	0.40, 0.86

Study country						
Philippines					*1.00*	
Indonesia					2.41***	1.54, 3.77
Vietnam					1.88*	1.11, 3.18
Log likelihood		−765.7427		−743.2761		−731.4547

OR = odds ratio; ***p < 0.001, **p < 0.01, *p < 0.05, ˆ*p* < 0.1.

## Footnotes

1 Information on disability was collected for each household member during household screening. The question asked whether the household member had ‘a permanent health problem that stops him/her from performing normal daily activities’. This was usually interpreted as referring to physical disability and neither of the variables ‘carer disability’ or ‘someone in household has disability’ is significantly correlated with carer CMD. Although we cannot be sure that there is no bias in the answers to the disability questions, the number of carers reported as having a disability is small (with only 3 cases in Vietnam) and the modelling results are substantially the same when the carer disability variable is dropped. Since the question was not always answered by the carer themselves, a category ‘unknown’ is included for this variable.

2 The diversity of the ‘Other-carer/Parent(s)-migrant’ group means that the significantly higher odds of CMD for carers in this group are difficult to interpret. Further investigation, which distinguished grandparents from other non-parental carers, revealed that Indonesian grandparent carers were more than twice as likely to suffer poor mental health (OR = 2.04, *p* < 0.05), whereas the difference between other non-parent carers and the comparison group is not significant. In the sample for the Philippines, both grandparents and other non-parental carers have higher odds of CMD but the results only approach significance (OR = 3.52, *P* < 0.1 and OR = 2.24, *p* < 0.1 respectively). Caution is needed in interpreting these results because of small numbers. Results for Vietnam, where the number of grandparent carers is higher, are not significant. Further research is therefore needed to ascertain whether or not grandparent carers in transnational households in Indonesia and the Philippines are particularly vulnerable to poor mental health.
